# Can There Be Drug Action without Drug Presence?

**Published:** 1892-01

**Authors:** 


### CAN THERE BE DRUG ACTION WITHOUT DRUG PRESENCE?

(Transactions of I. H. A., Session of Tuesday evening, June 23d, 1891.)

Dr. Rushmore having read a paper bearing the above title, the following discussion followed.

Dr. Fincke—The very finest and most delicate experiments that have been made by means of the spectroscope do not show anything higher than the eleventh or twelfth potency, but what is that against our 1M or our 1MM as long as we can watch and have positive evidence of the action of the same on the human organism, and for this reason I beg of you all to stand up for our homœopathic science. We are far beyond the commonly received science, and why should we not take the advanced position that belongs to us and to which we are entitled by the careful experiments of nearly a hundred years.

Dr. Biegler—It seems from the paper that we must still have matter in a potency to have any force in it ; or, in other words, there can be no force without some of the original matter being present.

Now I cannot believe that in the CM, for instance, there is any of the original substance left. I feel quite sure that the force is in existence, and that we administer not matter but force, and that we deal in forces. We are not using sledge hammers. I can believe that force exists without matter because I cannot conceive how force can be destroyed.

Dr. Cowley—I should like to ask Dr. Biegler why he cannot conceive of any matter being in the CM potency. I should like to hear his reasons.

Dr. Biegler—I do not deny the possibility of matter being in that potency, I merely say that my mind cannot comprehend how matter can exist in such a degree of attenuation ; it seems to me it must become dissipated. The matter may pass away but the force never.

Dr. Fincke—The burden of the proof does not lie on us. If matter is present it cannot be found out by any method of analysis. The chemists are all uniform in the claim that there is a limit to the divisibility of matter, and hence, if we pass beyond that limit in our potentiation, we must leave the matter behind. We cannot apply the force present in drugs hampered by the crude matter; which serves as the vehicle of force, not as its originator.

Hence you can see how the question of potency is inseparably attached to the main question of Homœopathy, and how important it is to cultivate the idea of the potencies.

Dr. Biegler—I do not wish to be understood as saying that we administer force without matter, but that we administer force without the matter that originally contained it.

Dr. Cowley—The claim of the chemists that there is a limit to the divisibility of matter is an assumption and nothing more. Might we not as well reconstruct the scientific theories of the day since we have more light than the mere materialists? Force is inherent in matter and cannot be separated from it. As one substance differs from another, so do the inherent forces differ. Each force requires its own peculiar vehicle, and will not inhere in any other. Hence, however attenuated, there must be particles of the original substance in the potency or else the force of that substance will not be there; it cannot be transferred to the milk sugar, for milk sugar cannot possibly be a vehicle for all drug forces. A potency can have no action unless there is drug presence.

Dr. Biegler—In the administration of remedies, the force of the remedy does not act on the material body but on the vital force, and I think that idea renders the assumption of drug presence unnecessary.

Dr. Wesselhœft—It seems to me that this is a matter that we cannot know much about, but we can and do know that the 1M potency acts. We cannot demonstrate how, it is one of the impossibilities. We did not know and could not demonstrate that there was any matter in the 11th potency before the use of the spectroscope. It makes no difference to me whether the presence of matter can be demonstrated or not, I am perfectly well satisfied of their action.

Dr. Rushmore—We must proceed according to certain forms. Can any one conceive of a formless force? No one can think of a force apart from some form, for the imagination will instantly clothe the idea with a form. Every force must have an appropriate form to act upon, for without that it is nothing. If the force in the potency has its form, can there be a form without the presence of the drug?

Dr. Stow—For the purpose of recreation I took a street-car ride, in Utica, and while riding I got to thinking about the wonderful force that propelled the car. It seemed to me that that was an illustration of the fact that force may exist apart from any material substance, and perform its work.

We can have no conception of the amount of matter in the CM potency; it is beyond human ideas and so is its form; but it is within the reach of a human being when applied to a deranged vitality. In other words, it is a force working apart from matter. But this question is abstract; we all agree on the curative effect of our medicines, let us not be divided because we cannot agree on the nature of spirit and matter.

Dr. Custis—No doubt we all can agree that power does exist in the potencies, but it seems to me that we cannot employ our time better than in discussing and studying these fundamental questions. I believe that force like matter is absolutely indestructible, and co-exists with matter. The germ of the oak is in the acorn, and the force there present is sufficient to draw together the atoms of carbon, oxygen, and hydrogen, and to arrange them into the form of a tree a hundred thousand times larger than the original kernel. Neither the microscope nor the spectroscope can find the germ in the acorn and yet it must be there.

